# The associations between adult body composition and abdominal adiposity outcomes, and relative weight gain and linear growth from birth to age 22 in the Birth to Twenty Plus cohort, South Africa

**DOI:** 10.1371/journal.pone.0190483

**Published:** 2018-01-16

**Authors:** Alessandra Prioreschi, Richard J. Munthali, Juliana Kagura, Rihlat Said-Mohamed, Emanuella De Lucia Rolfe, Lisa K. Micklesfield, Shane A. Norris

**Affiliations:** 1 MRC/WITS Developmental Pathways for Health Research Unit, Department of Paediatrics, School of Clinical Medicine, Faculty of Health Sciences, University of Witwatersrand, Johannesburg, South Africa; 2 MRC Epidemiology Unit, Institute of Metabolic Science, University of Cambridge School of Clinical Medicine, Cambridge Biomedical Campus, Cambridge, United Kingdom; State University of Rio de Janeiro, BRAZIL

## Abstract

**Background:**

The growing prevalence of overweight and obesity in low- or middle-income countries precipitates the need to examine early life predictors of adiposity.

**Objectives:**

To examine growth trajectories from birth, and associations with adult body composition in the Birth to Twenty Plus Cohort, Soweto, South Africa.

**Methods:**

Complete data at year 22 was available for 1088 participants (536 males and 537 females). Conditional weight and height indices were generated indicative of relative rate of growth between years 0–2, 2–5, 5–8, 8–18, and 18–22. Whole body composition was measured at year 22 (range 21–25 years) using dual energy x-ray absorptiometry (DXA). Total fat free soft tissue mass (FFSTM), fat mass, and abdominal visceral adipose tissue (VAT) and subcutaneous adipose tissue (SAT) were recorded.

**Results:**

Birth weight was positively associated with FFSTM and fat mass at year 22 (β = 0.11, p<0.01 and β = 0.10, p<0.01 respectively). Relative weight gain from birth to year 22 was positively associated with FFSTM, fat mass, VAT, and SAT at year 22. Relative linear growth from birth to year 22 was positively associated with FFSTM at year 22. Relative linear growth from birth to year 2 was positively associated with VAT at year 22. Being born small for gestational age and being stunted at age 2 years were inversely associated with FFSTM at year 22.

**Conclusions:**

The importance of optimal birth weight and growth tempos during early life for later life body composition, and the detrimental effects of pre- and postnatal growth restriction are clear; yet contemporary weight-gain most strongly predicted adult body composition. Thus interventions should target body composition trajectories during childhood and prevent excessive weight gain in early adulthood.

## Introduction

In low-or middle-income countries (LMIC), overweight and obesity are becoming more prevalent in males and females of all age ranges and socio-economic strata [1]. Furthermore, there is a double burden of malnutrition with overweight and obesity developing within the context of early life malnutrition [2, 3]. A recent South African national survey reported that in children, one fourth of those who are under 3 years of age were stunted; and the highest prevalence of overweight and obesity (18.1% and 4.6%, respectively) was found in children between 2 and 5 years of age [1]. Longitudinal studies have consistently linked obesity during childhood and adolescence with increased risk of cardiovascular disease and all cause mortality in adulthood [4, 5].

The Developmental Origins of Health and Disease (DOHaD) hypothesis suggests that pre and postnatal under- or overnutrition may alter organ structures and physiological functions, resulting in changes that increase future disease risk, including higher levels of adiposity and its abdominal repartition [6, 7]. Birth weight has been widely used as an indicator of prenatal growth, and has been associated with growth and development, as well as mortality due to cardiovascular and metabolic diseases [8]. A recent meta-analysis reported a positive association between birth weight and overall body size (waist and hip circumferences), but that the association between birth weight and central distribution of fat mass (waist-to-hip ratio) was largely confounded by adult body mass index (BMI) [9]. Accordingly, the authors suggested that birth weight may predict overall adult size, but that postnatal growth may play a more important role in determining adult body fat distribution. Consequently, shifting the focus to body composition has resulted in consistent evidence for positive and stronger associations between birth weight and fat free mass rather than fat mass [10, 11].

Postnatal growth trajectories in infancy and childhood have been reported to be associated with adult disease risk [12, 13]. A significant limitation in available studies is the use of anthropometric indicators of adiposity and central body fat [9, 10, 14, 15], which limits the ability to assess the relationships between pre and postnatal growth and more specific measures of later body composition. In addition, the paucity of longitudinal data from LMICs limits the opportunities to assess these relationships beyond the infancy and childhood periods. Therefore, the aim of the current study was to assess the association between growth in weight and height from birth to adulthood, and body composition and abdominal adiposity in adulthood (at 22 years of age) measured by DXA; and to assess the relationships between IUGR, infant under- and overnutrition (stunting and overweight) at age 2 years, and body composition and abdominal adiposity at 22 years of age in the urban Birth to Twenty Plus (Bt20+) cohort in South Africa.

## Methods

### Study population

This study sample was drawn from the BT20+ cohort study in Soweto, South Africa. The original cohort (n = 1594) recruited and followed up all singleton children born to women living in Soweto from April 1990, with the intention of recruiting a cohort of urban children representative of long-term residents of Johannesburg/Soweto. Data was collected on a number of variables, including birth weight, growth, socioeconomic status, and maternal factors. The details of this cohort have been described elsewhere [16]. At year 22, participants who were free from any congenital disorders or deformities were invited back for a follow up visit, which comprised of various anthropometric measurements [17]. All participants provided informed consent, and ethical approval for these studies was obtained from the Human Research Ethics Committee of the University of the Witwatersrand, South Africa (24/1/90, M01-05-56 bt20, M111182).

### Exposures

#### Birth weight, and conditional weight and height measures

Birth weight was obtained from participants’ birth records, and was measured by hospital staff according to standard procedures. Intra-uterine growth restriction was estimated using small for gestational age (SGA), which was defined as birth weight for gestational age and sex below the 10^th^ percentile [18]. Each subsequent measurement of weight was measured by trained research staff to the nearest 0.1kg using a digital scale (Dismed, USA) according to standardised procedures. Conditional weight gain was generated by regressing the weight at each age on all previous weight and height measures adjusting for current height as described previously [19], thereby indicating the relative rate of weight gain since the preceding time point. Trained research staff measured height during infancy to the nearest 1mm using an infantometer, according to standardised procedures. Thereafter, height was measured to the nearest 1cm using a wall-mounted stadiometer (Holtain, UK). To deal with bias due to repeated weight and height measures, we calculated conditional height gain by regressing the height at each time point on all previous height and weight measures thus generating conditional height variables [20]. Previous research in this cohort [21] has shown that age of pubertal initiation ranges between 9.8 to 10.5 years; and therefore we considered year 8 to be pre-pubertal. Growth periods were thus years 0–2 (early life), years 2–5 (mid childhood), years 5–8 (pre-pubertal), years 8–18 (peri- and post-pubertal), and years 18–22 (young adult). Height and weight measured at each time point were used to calculate BMI (weight(kg)/height(m)^2^). Overweight at age 2 was defined as a BMI for age z-score above +2 at year 2, and stunting was defined as a height for age z-score below -2 at year 2 according to WHO growth standards [18].

### Outcome variables

#### Fat free soft tissue mass, fat mass, abdominal VAT and SAT at age 22

Whole body composition was measured at year 22 using a Hologic QDR 4500A DXA machine and analysed using Apex software version 4.0.2 (Hologic Inc., Bedford, USA). All scans were performed according to standard procedures by a trained technician. The machine was calibrated daily using a phantom spine, and coefficients of variation during the course of the study were <2% for total fat mass, and 1% for fat-free soft tissue mass. All standard DXA measurements were analysed using Hologic APEX 3.1 software (Hologic). Whole body (excluding head) fat free soft tissue mass (FFSTM) and fat mass were recorded. Abdominal visceral adipose tissue (VAT) and subcutaneous adipose tissue (SAT) were measured according to previously described methodology [22].

### Other variables

Data on *a priori* potential confounders were drawn from the original cohort data. Descriptions on the collection of these data are described elsewhere [16]. Confounders used in the analysis included socioeconomic status (SES) measured at year 22 (which comprised of a sum of assets score), gestational age, ethnicity, and maternal height.

### Statistical analysis

All analyses were conducted using STATA 13 (StataCorp, College Station, TX, USA). All data are presented as mean (SD) for continuous data, or percentages for categorical data. All females who were pregnant during data collection were excluded from analyses. Students unpaired t-tests and chi-squared tests were used to compare differences between males and females for continuous and categorical data, respectively. Body composition variables were log transformed (SAT, FFSTM, and fat mass) or square-root transformed (VAT) if not normally distributed, and coefficients presented are therefore transformed estimates. Multiple linear regressions were run to determine the associations between birth weight and conditional weights with each of the following outcomes separately: fat mass, FFSTM, VAT, and SAT at year 22; as well as for conditional heights with the same outcomes separately. Multiple linear regressions were used to determine associations between the categorical variables stunting at age 2, overweight at age 2, and small for gestational age with the outcome measures separately. All regressions were adjusted for *a priori* confounders as mentioned above. Significance was set at p<0.05 in all instances.

## Results

Complete DXA data at year 22 was available for 1088 participants (536 males and 537 females). This sample was not different from the original cohort in terms of birth factors or maternal demographics (see S1 Table), but gestational age was higher, and SES was lower (p<0.01 in both cases) in the original cohort. Furthermore, the percentage of Black participants was higher in the included sample (p<0.01), although this was due to the recruitment focus at year 22. All of these factors were controlled for in subsequent regression analyses. Anthropometric data for the included participants (if available) at birth, year 2, year 5, year 8, year 18, and year 22 are shown in Table 1. The mean (range) age at each time point of data collection were as follows: 1.6 (1.0–2.4) years; 4.8 (4.0–5.6) years; 8.3 (7.2–9.0) years; 17.1 (16.0–18.0) years; and 23.0 (21.0–24.5) years respectively.

**Table 1:** Participant characteristics from birth to age 22 in all participants, combined and by sex. All values are mean (SD) or n(%).

	All			Males			Females			
Measure/Age	N	Mean	Std. Dev.	N	Mean	SD	N	Mean	SD	p value
Birth weight (kg)	1071	3.1	0.5	534	3.1	0.5	537	3.0	0.5	**<0.001**
Weight at 2 years (kg)	610	10.5	1.7	300	10.7	1.6	310	10.4	1.7	**0.015**
Weight at 5 years (kg)	700	17.2	2.5	348	17.3	2.4	352	17.1	2.6	0.275
Weight at 8 years (kg)	606	25.0	4.1	305	24.6	3.6	301	24.4	4.6	0.547
Weight at 18 years (kg)	1014	57.6	10.7	514	58.0	10.1	500	57.1	11.3	0.210
Weight at 22 years (m)	1032	63.6	13.2	520	63.5	11.4	512	63.8	14.8	0.708
Height at 2 years (cm)	608	78.7	5.5	297	79.2	5.3	311	78.2	5.7	0.024
Height at 5 years (cm)	700	104.8	5.9	348	104.7	5.8	352	104.9	5.9	0.699
Height at 8 years (kg)	608	124.1	5.9	307	124.3	5.8	301	123.9	6.0	0.324
Height at 18 years (cm)	1015	164.4	8.2	514	169.6	6.7	501	159.0	5.9	**0.000**
Height at 22 years (m)	1039	165.5	8.7	521	171.5	6.4	518	159.5	6.1	**0.000**
BMI at 8 years (kg)	606	15.8	1.8	305	15.9	1.5	301	15.8	2.1	0.811
BMI at 18 years (kg/m^2^)	1014	21.3	3.9	514	20.1	3.1	500	22.6	4.3	**0.000**
BMI at 22 years (kg/m^2^)	1032	23.3	5.0	520	21.6	3.6	512	25.1	5.7	**0.000**
VAT (cm) at 22 years	1088	0.50	0.30	536	0.47	0.22	537	0.60	0.37	**0.000**
SAT (cm) at 22 years	1088	2.00	1.60	536	0.98	0.83	537	0.31	0.15	**0.000**
FFSTM (kg) at 22 years	1088	38.70	7.80	536	44.12	5.98	537	33.25	5.23	**0.000**
Fat mass (kg) at 22 years	1088	18.10	10.50	536	12.01	6.42	537	24.12	10.22	**0.000**
Asset based-SES score at 22 years	1073	9.6	2.6	536	9.5	2.6	537	9.7	2.6	0.230
Gestational age (weeks)	1056	38.0	2.0	529	38.1	1.9	527	37.9	2.0	0.167
Maternal education (schooling years)	991	9.6	2.7	493	9.4	2.7	498	9.8	2.7	**0.033**
Maternal Age (years)	1069	26.0	6.3	535	26.1	6.3	534	26.0	6.2	0.773
Maternal Height (cm at trimester 3)	1020	158.4	6.5	509	158.6	6.6	511	158.2	6.3	0.330
Small for Gestational age (%)										
*No*	921(87.4)			452(86)			469(89)			0.115
*Yes*	133(12.6)			75(14)			58(11)			
Stunted age 2 (%)										
*No*	331(83.0)			164(82)			167(84)			0.610
*Yes*	68(17)			36(18)			32(16)			
Overweight age 2 (%)										
*No*	338(85)			168(84)			170(85)			0.692
*Yes*	61(15)			32(16)			29(15)			
Ethnicity (%)										
Black	950(88.5)			478(89)			472(88)			0.586

Associations between relative linear growth, relative weight gain, and the body composition and abdominal adiposity outcomes at year 22 are presented in Table 2. A sample of 266 participants had complete DXA data at year 22, as well as complete growth data at each timepoint for inclusion in the final analysis. Relative weight gain for each of the time periods from birth to year 22 was positively associated with FFSTM, fat mass, VAT, and SAT at year 22. Furthermore, birth weight was positively associated with FFSTM and fat mass at year 22. Relative linear growth for each of the time periods from birth to year 22 was positively associated with FFSTM at year 22, while relative linear growth from birth to year 5 (0–2 years and 2–5 years) was positively associated with fat mass at year 22; and from birth to year 2 was positively associated with VAT at year 22. Relative linear growth from years 18–22 was positively associated with fat mass, VAT and SAT at year 22.

**Table 2:** Multiple linear regression analysis of relative weight gain and relative linear growth on adult FFSTM, fat mass, SAT and VAT.

Relative weight gain					Relative linear growth			
	beta	95%CI		P value	beta	95%CI		P value
**FFSTM (n = 266)**					**FFSTM (n = 267)**			
Birth	0.105	0.075	0.135	**<0.001**				
0–2 years	0.026	0.013	0.038	**<0.001**	0.026	0.011	0.041	**0.001**
2–5 years	0.039	0.026	0.052	**<0.001**	0.036	0.021	0.051	**<0.001**
5–8 years	0.028	0.016	0.041	**<0.001**	0.040	0.025	0.055	**<0.001**
8–18 years	0.043	0.031	0.055	**<0.001**	0.045	0.029	0.060	**<0.001**
18–22 years	0.058	0.047	0.070	**<0.001**	0.036	0.020	0.051	**<0.001**
**Fat mass (n = 266)**					**Fat mass (n = 267)**			
Birth	0.096	0.028	0.165	**0.006**				
0–2 years	0.069	0.041	0.097	**<0.001**	0.044	-0.008	0.096	**0.096**
2–5 years	0.156	0.126	0.185	**<0.001**	0.062	0.010	0.114	**0.019**
5–8 years	0.105	0.077	0.134	**<0.001**	0.042	-0.008	0.092	0.103
8–18 years	0.182	0.155	0.210	**<0.001**	0.010	-0.043	0.062	0.717
18–22 years	0.231	0.204	0.257	**<0.001**	0.121	0.069	0.173	**<0.001**
**SAT (n = 266)**					**SAT (n = 267)**			
Birth	0.085	-0.026	0.197	0.133				
0–2 years	0.069	0.024	0.114	**0.003**	0.041	-0.033	0.114	0.275
2–5 years	0.211	0.162	0.259	**<0.001**	0.052	-0.021	0.124	0.161
5–8 years	0.138	0.092	0.184	**<0.001**	0.038	-0.032	0.109	0.285
8–18 years	0.243	0.198	0.288	**<0.001**	-0.010	-0.083	0.064	0.793
18–22 years	0.297	0.254	0.341	**<0.001**	0.178	0.104	0.251	**<0.001**
**VAT (n = 266)**					**VAT (n = 267)**			
Birth	-0.109	-0.542	0.324	0.621				
0–2 years	0.236	0.060	0.412	**0.009**	0.270	0.023	0.518	**0.032**
2–5 years	0.583	0.396	0.771	**<0.001**	0.063	-0.183	0.308	0.616
5–8 years	0.385	0.207	0.564	**<0.001**	-0.110	-0.348	0.127	0.361
8–18 years	0.650	0.476	0.824	**<0.001**	-0.028	-0.276	0.221	0.827
18–22 years	0.935	0.767	1.102	**<0.001**	0.367	0.120	0.614	**0.004**

All regressions are controlled for gestational age, ethnicity, socioeconomic status, maternal height, and sex

Sensitivity analyses showed some sex differences. Birth weight remained significantly associated with fat mass at year 22 in males only (β = 0.13, p = 0.02). Relative weight gain between years 0–2 remained significantly associated with VAT and SAT at year 22 in females only (in males: β = 0.16, p = 0.09; β = 0.07, p = 0.05 respectively). When stratified by sex, associations between relative linear growth and FFSTM at year 22 remained significant between years 0–2 and years 8–18 for both sexes, but were no longer significant between years 5–8 in males (p = 0.06); and between years 2–5, 5–8, and 18–22 in females (p = 0.11, p = 0.27, p = 0.44 respectively). Relative linear growth between years 2–5 remained significantly associated with fat mass at year 22 in females only, and between years 18–22 remained significant with fat mass at year 22 in males only. For females, relative linear growth between years 2–5, and years 8–18 was significantly associated with SAT at year 22 (p = 0.03 and p = 0.04 respectively), while for males relative linear growth between years 0–2 (p = 0.01) was significantly associated with SAT at year 22. Associations with relative linear growth between years 18–22 and SAT at year 22 were no longer significant for females (β = -0.07, p = 0.17). Lastly, associations with relative linear growth between years 18–22 and VAT at year 22 remained significant in males only, while associations with relative linear growth between years 0–2 and VAT at year 22 were no longer significant for either sex.

Associations between stunting and overweight at age 2, and IUGR with body composition in early adulthood were tested. Intrauterine growth restriction (IUGR) and being stunted at age 2 were negatively associated with FFSTM at year 22 (IUGR: β = -0.04, CI = -0.07; -0.01, p<0.01; stunted: β = -0.07, CI = -0.11; -0.04, p<0.01). Overweight at age 2 was positively associated with FFSTM and fat mass at year 22 (FFSTM: β = 0.08, CI = 0.04; 0.12, p<0.01 and fat mass: β = 0.21, CI = 0.09; 0.34, p<0.01), as well as with VAT and SAT at year 22 (VAT: β = 0.65, CI = 0.10; 1.20, p = 0.02 and SAT: β = 0.24, CI = 0.06; 0.41, p<0.01).

## Discussion

This study has shown results similar to those from meta-analyses and international LMIC birth cohorts (Brazil, Guatemala, India, Philippines, and South Africa) [9, 23, 24]. The use of imaging techniques for measurement of body composition has however allowed for better determination of lean and fat mass, abdominal adipose tissue depots, and their associations with relative growth. Relative weight gain from birth to adulthood was associated with all favourable and unfavourable body composition outcomes at year 22; yet this relationship was stronger for adipose tissue accumulation and became stronger as age increased. This implies that contemporary weight gain remained the most important predictor of adult body adiposity. Furthermore, stunting and IUGR were associated with decreased lean body mass, while overweight at age 2 was positively associated with all adult body composition measures, including lean body mass. Thus, both under- and over-nutrition were shown to negatively impact adult body composition, but growth restriction was associated with more deleterious effects.

A systematic review by Rogers et al has shown birth weight to be associated with central adiposity [10], however few studies have used imaging techniques to measure body composition and body fat distribution in order to confirm these findings [9]. Previous birth cohort studies have only shown associations between birth weight and adult BMI and adiposity [9, 23], yet information on the distribution of this adiposity has been more limited. The Pelotas birth cohort study recently examined associations between relative growth and abdominal adiposity measured using ultrasound imaging techniques in adults [24]. They showed that relative weight gain during early childhood (2–4 years), later childhood and adolescence (4–23 years), and adulthood (23–30 years) were all associated with visceral and subcutaneous fat at age 30, and that these associations strengthened with age. The findings from the present study concur; no associations were noted between birth weight and adult abdominal adiposity in either the Pelotas or the current cohort study. However, in the current study, both birth weight (in males specifically) and relative weight gain at all time points were associated with increased fat mass at year 22, as was relative linear growth at all timepoints except for between years 5–8. Importantly, growth between birth to year 2 was more strongly associated with VAT in adulthood than with other body composition outcomes; yet when stratified by sex this relationship (and that with SAT at year 22) remained for females only. Since associations with birth weight were not supported by the abdominal adiposity measures, these data may suggest that previous studies showing associations with birth weight and infancy weight gain on BMI could have been reflecting associations with overall adult size, rather than with abdominal adiposity. Although still requiring further investigation, some studies have shown that increased relative growth early in life may also be associated with adult metabolic complications [25]; and associations seen in this study with adult adiposity may support these findings. However, since adult lean mass was also concurrently associated (albeit more weakly), relative growth in height and weight may in fact be causing increased adult size in totality.

In previous studies conducted in LMICs, rapid weight gain in childhood (after the age of two) has been associated with greater adiposity, while early (between 0–2 years) rapid weight gain has been associated with lean body mass [14, 23, 26–28]. Later rapid weight gain is thought to be more deleterious to adult health than early rapid weight gain [25, 29], since it usually results in increases in fat mass (particularly central adiposity) with no change in height i.e.: ‘stunted-obese’ individuals. This is particularly evident in LMICs, where the prevalence of stunting and malnutrition is high, and children are thus often born smaller, and may become growth restricted during infancy or childhood, resulting in later rapid catch up growth [25]. Indeed, in the present study, 13% of participants were born small for gestational age, and 17% were stunted at age 2, while at the same time 15% were overweight at age 2 –highlighting the double burden of over- and under-nutrition in this context. However, in the current study, both early linear growth independent of weight (between years 0–2) and later relative weight gain (at each timepoint between years 2–22) were associated with increased abdominal adiposity, as well as, with fat mass and lean mass. It is thus important to note that in all cases, associations with adult adiposity became stronger as age increased–with contemporary weight gain (years 18–22) being the most strongly associated with adult body composition and abdominal adiposity. Childhood and adolescent obesity has been linked to adult cardiovascular health and mortality in large-scale longitudinal studies [4, 5]. Thus, while the childhood period is important in establishing body composition trajectories and metabolic programming for adult health, the early adulthood period is still a crucial period of life to target for lifestyle modifications aimed at prevention of poor adiposity outcomes in adulthood.

In the present study, lean mass in young adulthood was determined by both relative weight gain and linear growth from birth to year 22. Conversely, being born SGA, or being stunted at age 2 were both inversely associated with lean mass in adulthood (likely as a result of the preferential restriction of skeletal muscle growth that occurs during growth restriction [30]). Decreased adult lean mass has been associated with insulin resistance, metabolic syndrome, Type 2 diabetes, and cardiovascular disease [30]. The current findings suggest that rapid growth (weight and length) during infancy and childhood were beneficial for adult lean mass outcomes; however that growth restriction and stunting during early life negatively impacted lean mass in adulthood, regardless of potential catch up growth which may have occurred thereafter. In fact interaction analyses showed that in those who were growth restricted (SGA or stunted at age 2), rapid weight gain at any time point was no longer associated with adult lean mass. These findings are in line with previous studies showing that if growth restriction has occurred, fat mass is preferentially accumulated, resulting in decreased adult lean mass [30]. It must however be noted that the sample of growth restricted individuals was too small to provide enough power for a regression analysis (n = 24).

Linear growth retardation has been shown to be associated with decreased adult lean mass in previous studies [25, 28], and our findings corroborated this as we showed that being stunted at age 2 was negatively associated with adult lean mass. There is controversy in the literature around the association between stunting and later fat mass. In the present study rapid linear growth in the first five years of life was associated with increased fat mass (as well as lean mass), and rapid linear growth in the first two years was associated with increased visceral adiposity (and with increased abdominal subcutaneous fat in males). This study may therefore address some of the controversies in the literature, by providing imaging measurements of body composition and abdominal adiposity distribution. The current findings indicate that, while being stunted at age two has detrimental effects on adult lean mass, rapid linear growth in early life could also be detrimental to adult body composition, resulting in increased adiposity in conjunction with increased lean mass. Furthermore, in the present study, being overweight at age two was associated with higher lean and fat mass, as well as with abdominal adiposity. Bearing in mind that these regressions were conducted on only 15% of the sample, it seems that in this setting both growth restriction (pre and postnatally), and excess weight gain and linear growth in early childhood had detrimental effects on adult body composition and could thus potentially impact later health.

Limitations of this study include the lack of a measure of length at birth, making it impossible to differentiate prenatal from postnatal linear growth effects. Strengths of the current study include the use of imaging techniques to measure body composition and abdominal adipose depots; as well as controlling for factors such as gestational age, socioeconomic status, ethnicity and maternal height. Furthermore, the use of conditional weight and height, although it resulted in a decreased sample size, allowed us to avoid collinearity within the regression models, while still controlling for current and previous body size [23]. The applicability of our results to current South Africa and to other black populations is a limitation, yet the cohort provides a good representation of long-term residents of urban Johannesburg/Soweto at the time of delivery in 1990. All regression models were adjusted for any differences between the study sample and the participants with missing data, yet due to the differences that did exist imputation was not considered, since assumptions about the nature of the missing data could not be made. Lastly, we have been able to differentiate between early life, prepubescent, pubescent childhood, and adolescent growth associations, which has not been done in other LMICs.

In conclusion, findings from this longitudinal birth cohort have emphasised the importance of optimal early life growth in determining later body composition, by highlighting the risks of pre- and postnatal growth restriction for later life health. Greater relative growth in early- and mid-childhood, as well as in pre- and post-pubescent periods result in increased adult body size through increases in all body composition compartments, but particularly fat deposition. Greater relative weight gain in adulthood (years 18–22) was most strongly associated with worse adiposity outcomes. This study has added to the current literature by confirming some findings from developed countries, and describing the contrasting patterns that occur in LMICs where growth restriction remains a key public health issue. Furthermore, we have shown associations with relative weight gain and adult abdominal depots, and some differential timing effects between sexes. This study highlights the need to intervene effectively during early childhood by preventing growth restriction or excessive weight gain, but also the importance of preventing excessive weight gain in early adulthood in order to prevent adult obesity and related co-morbidities.

## Supporting information

S1 Table Comparison of sociodemographic profiles of participants in the study sample and excluded sample. (DOCX)
