# Supplementary material for: The associations between adult body composition and abdominal adiposity outcomes, and relative weight gain and linear growth from birth to age 22 in the Birth to Twenty Plus cohort, South Africa
Source: PLoS One. 2018 Jan 16;13(1):e0190483. doi: 10.1371/journal.pone.0190483 (PMC5770024; doi:10.1371/journal.pone.0190483)
Supplement: S1 Table — (DOCX) [file pone.0190483.s001.docx]

| **Variable** | **Study sample** | **Original Cohort** | **P value** |
| --- | --- | --- | --- |
| Maternal age (years) | 26.0(6.3) | 26.0(6.0) | 0.74 |
| Maternal schooling (years) | 9.6(2.7) | 9.5(3.1) | 0.81 |
| Birth weight (kg) | 3.1(0.5) | 3.1(0.5) | 0.84 |
| Gestational age (weeks) | 37.9(2.0) | 38.2(1.9) | <0.01 |
| Socioeconomic status at 22 years (n/13) | 9.6(2.6) | 9.2(3.1) | <0.01 |
| Sex (%) |  |  |  |
| Males | 536(50.0) | 1115(47.9) | 0.21 |
| Females | 535(49.9) | 1230(52.3) |  |
| Small for gestational age (%) |  |  |  |
| No | 921(87.4) | 1794(84.9) | 0.06 |
| Yes | 133(12.6) | 320(15.1) |  |
| Ethnicity (%) |  |  |  |
| Black | 950(88.5) | 1625(69.9) | <0.01 |
| Other | 123(11.5) | 701(30.1) |  |

**S1 Table: Comparison of sociodemographic profiles of participants in the study sample and excluded sample**
